# Detection rate of [^99m^Tc]Tc-PSMA SPECT/CT in prostate cancer: a systematic review and a meta-analysis

**DOI:** 10.3389/fmed.2026.1827510

**Published:** 2026-04-13

**Authors:** Domenico Albano, Slavko Tasevski, Ramin Sadeghi, Giorgio Treglia, Alessio Rizzo

**Affiliations:** 1 Department of Nuclear Medicine, University of Brescia, Brescia, Italy; 2 Department of Nuclear Medicine, ASST Spedali Civili di Brescia, Brescia, Italy; 3 University Institute for Positron Emission Tomography, Skopje, North Macedonia; 4 Nuclear Medicine Research Center, Mashhad University of Medical Sciences, Mashhad, Iran; 5 Division of Nuclear Medicine, Imaging Institute of Southern Switzerland, Ente Ospedaliero Cantonale, Bellinzona, Switzerland; 6 Faculty of Biomedical Sciences, Università della Svizzera Italiana, Lugano, Switzerland; 7 Faculty of Biology and Medicine, University of Lausanne, Lausanne, Switzerland; 8 Department of Nuclear Medicine, Candiolo Cancer Institute, FPO-IRCCS, Turin, Italy

**Keywords:** meta-analysis, nuclear medicine, prostate cancer, PSMA, SPECT/CT

## Abstract

**Background/objective:**

Prostate-specific membrane antigen (PSMA) positron emission tomography/computed tomography (PET/CT) remains a fundamental diagnostic tool for Prostate cancer (PCa), yet its use is frequently constrained by the high costs and infrastructure requirements of PET imaging. [^99m^Tc]Tc-PSMA single photon emission computed tomography/CT (SPECT/CT) emerges as a viable diagnostic option, showing significant potential in both the primary staging and restaging of prostate cancer. We performed a systematic review and meta-analysis about the detection rate (DR) of [^99m^Tc]Tc-PSMA SPECT/CT in PCa patients.

**Methods:**

A comprehensive literature search of the PubMed/MEDLINE, Scopus, and Cochrane libraries was performed to extract relevant published articles about the DR of [^99m^Tc]Tc-PSMA SPECT/CT in patients affected by PCa.

**Results:**

Twenty-three studies (*n* = 1,840 patients) were included in the systematic review with 19 studies eligible for the meta-analysis. The pooled estimated DR of [^99m^Tc]Tc-PSMA SPECT/CT in the staging field was 79.7% (95% CI 70.2–89.3%); in restaging biochemical recurrence was 75.4% (95% CI 67.7–83%). A significant statistical heterogeneity was reported in both analyses. PSA value at the time of SPECT/CT was frequently associated with the DR.

**Conclusions:**

In conclusion, our analysis showed that [^99m^Tc]Tc-PSMA SPECT/CT is a good diagnostic tool for prostate cancer, both at staging and biochemical recurrence. It may be useful as an alternative in resource-constrained environments or remote locations where PET/CT technology is not readily accessible.

## Introduction

Prostate cancer (PCa) is the second most common type of cancer diagnosed in men and the fifth leading cause of death (1). The incidence varies substantially between countries, which is projected to increase in the following decades due to early detection and implementation of screening programs. The mortality rate could rise in some countries as well, due to limited availability of effective treatments (2). Different imaging procedures play a key role in early detection of PCa in patients with rising prostate specific antigen (PSA), as well as in guiding biopsy, determining the stage of disease and treatment management.

Prostate-specific membrane antigen (PSMA) ligand PET/CT imaging has become an important diagnostic tool for both localized and advanced PCa. PSMA is a type II transmembrane glycoprotein expressed in prostatic epithelium and markedly overexpressed in prostate cancer cells, particularly in high-grade disease, while showing limited expression in most normal tissues (3). Several PSMA-targeting radiotracers labeled with cyclotron-produced radionuclides like ^18^F-PSMA-1007, or generator-produced 68Ga like [^68^Ga]Ga-PSMA are currently used in clinical practice (4, 5). ^18^F tracers require cyclotron access, while ^68^Ga tracers rely on generator availability, making tracer selection dependent on local infrastructure. Each approach has advantages and limitations in terms of diagnostic performance, cost, and availability of cyclotrons/generators (6, 7). Consequently, the choice of tracer often depends on the infrastructure and resources available within a given healthcare system (8). Despite the established role of PSMA PET/CT, access remains limited in many regions.

Technetium-99m (^99m^Tc) is the most commonly used radionuclide in clinical nuclear medicine due to its broad availability, low radiation burden, favorable physical characteristics and lower cost compared to positron emitters. Several [^99m^Tc]Tc-PSMA based radiopharmaceuticals have been developed, including [^99m^Tc]Tc-EDDA/HYNIC-iPSMA, [^99m^Tc]Tc-MIP-1404, and [^99m^Tc]Tc-PSMA-I&S. Crucially, these compounds represent a heterogeneous class of tracers; differences in their chelating moieties and chemical linkers result in varied pharmacokinetic properties and biodistribution patterns. Therefore, while they share the same molecular target, their individual diagnostic performance and lesion-to-background ratios may not be entirely equivalent—a factor that must be carefully considered when interpreting pooled clinical data. From a cost-effectiveness perspective, these tracers may represent a valuable alternative in low or middle-income countries, particularly where access to PET/CT scanners, 68Ge/68Ga generators, or cyclotrons is limited (9).

The role of [^99m^Tc]Tc-PSMA in the management of prostate cancer has been investigated using SPECT/CT. Evaluating the diagnostic performance of this tracer is essential for defining its clinical utility and potential integration into routine practice. Therefore, we performed a systematic review and meta-analysis of published studies to determine the diagnostic accuracy and clinical value of ^99m^Tc-PSMA imaging in patients with prostate cancer.

## Methods

### Protocol

A direct review query using the Population, Intervention, Comparator, and Outcomes (PICO) framework was done: “What is the diagnostic role (“outcome”) of [^99m^Tc]Tc-PSMA SPECT/CT (“intervention”) in patients with PCa (“population”) compared with other imaging modalities or evaluated against reference standards (“comparator”).” Two investigators (D.A. and S.T.) independently performed the literature search, the study selection, the data extraction and the quality evaluation. In case of disagreements, a third opinion (G.T.) was asked.

### Search strategy

A comprehensive literature search of the PubMed/MEDLINE, Scopus, and Cochrane libraries was performed to extract relevant published articles about the detection rate (DR) of [^99m^Tc]Tc-PSMA SPECT/CT in patients affected by PCa. Moreover, a specific research on the ClinicalTrials.gov database for ongoing investigations (access date: 1 January 2026) was executed.

We used a search algorithm based on a combination of the following terms: (1) “[^99m^Tc]Tc-PSMA” OR “HYNIC-PSMA” AND (2) “prostate” OR “prostatic”.

No beginning date limit was used for our literature search, which was updated until December 1, 2025. Only articles in the English language were selected. To enlarge our research, references of the retrieved articles were also screened for searching additional papers. For the management of these articles, we used EndNote Basic (Thompson Reuters).

### Study selection

Studies or subsets of studies investigating the DR of [^99m^Tc]Tc-PSMA SPECT/CT in patients with PCa were eligible for inclusion in the qualitative (systematic review) and quantitative analysis (meta-analysis). The exclusion criteria for the systematic review were: (a) articles not within the field of interest of this review; (b) review articles, editorials or letters, comments, conference proceedings; (c) case reports or small case series (less than 10 patients with PCa included). For the meta-analysis, articles with possible patient data overlap were excluded; in this case, articles with more complete information were included in the meta-analysis. To ensure the independence of the data, a systematic screening for patient overlap was performed. We identified studies published by the same research groups or institutions and compared their recruitment time frames and inclusion criteria.

Titles and abstracts were independently reviewed by two researchers (S.T. and D.A.) applying the selected inclusion and exclusion criteria. Disagreements were solved in a consensus meeting.

### Data extraction and collection

For each research, several pieces of information were collected in particular about basic study characteristics (name of first authors, publication year, country of origin, funding source, and study design), features of patients included (mean/median age, mean/median PSA serum values, and setting). Furthermore, data on technical aspects were extracted (type of radiotracer, kind of scanner, radiotracer mean injected activity, uptake time, protocol of acquisition, other modalities performed for comparison). For articles included in the meta-analysis, data about DR values of [^99m^Tc]Tc-PSMA SPECT/CT was collected on a per-patient analysis in two different settings: staging and restaging of biochemical recurrence. To avoid potential biases, the researchers separately gathered each of the studies and extracted data from the information in the entire manuscript, figures, and tables.

The main findings of the articles included in this review were represented in Tables and in the “Results” section.

### Quality assessment (risk of bias assessment)

A quality assessment of included articles was performed to analyze the risk of bias in individual studies to the review query. Four domains (patient selection, index test, reference standard, and flow and timing) were evaluated for risk of bias. At the same time, three sectors were assessed for applicability concerns (patient selection, index test, and reference standard) by using the QUADAS-2 tool (10).

### Statistics

DR was defined as the ratio between the number of scans with at least one suspected lesion detected and the total number of scans performed. Pooled analysis about DR was performed using data retrieved from the included studies. A random-effects model (as suggested by DerSimonian and Laird) was used for the pooled analysis (11). Pooled estimates and related 95% confidence interval (95% CI) values were calculated. Forest plots were provided for the meta-analysis. I-square index (I2) or inconsistency index was used to estimate the statistical heterogeneity (12), whereas the publication bias was assessed through the visual analysis of the funnel plot and using Egger's test (13). For this analysis, we used open-source OpenMetaAnalyst software developed by the Center for Evidence Synthesis in Health of Brown University (USA) for the statistical analysis.

## Results

### Literature search

**Figure 1:**
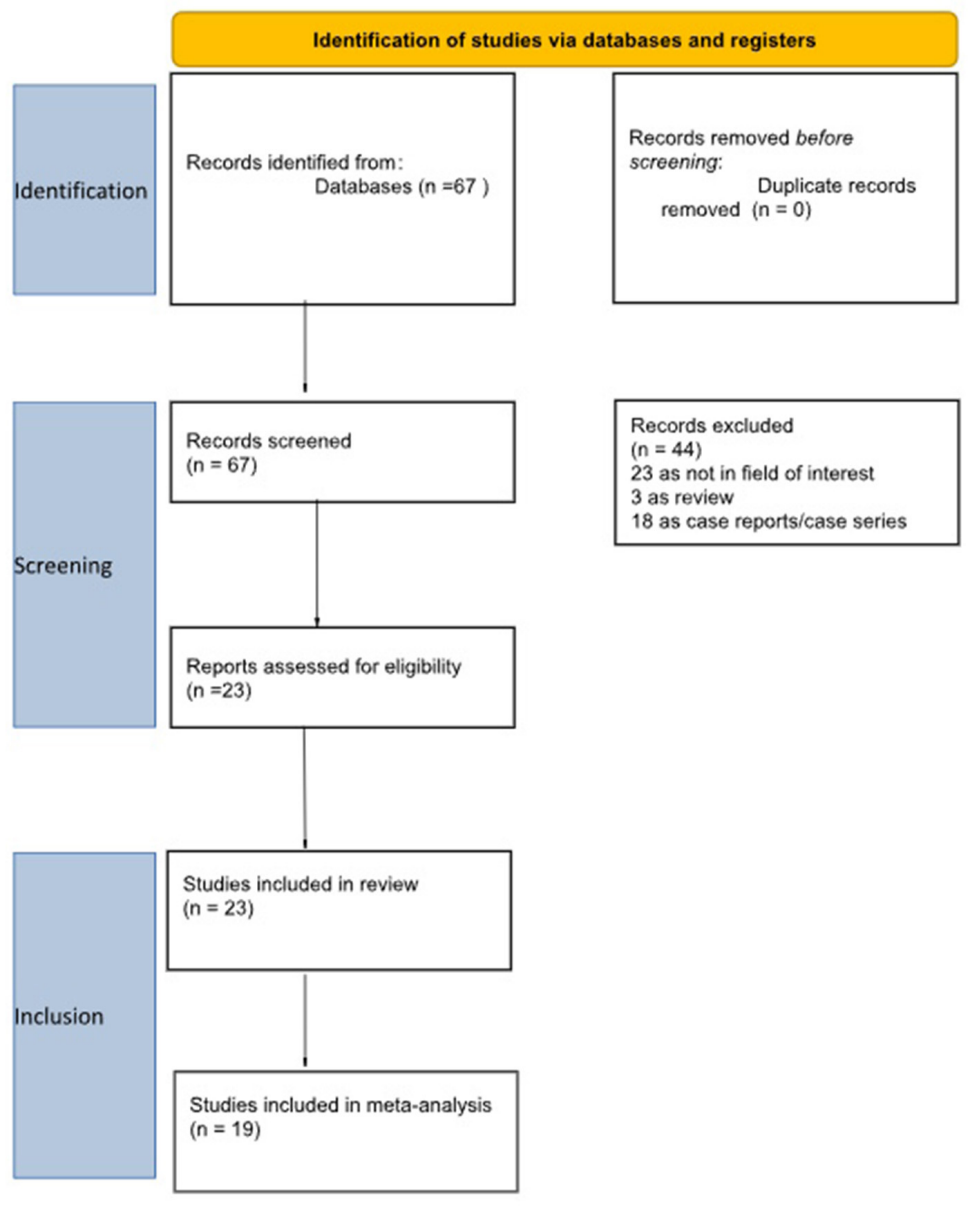
Flow-chart of the selection of the articles.

**Figure 2:**
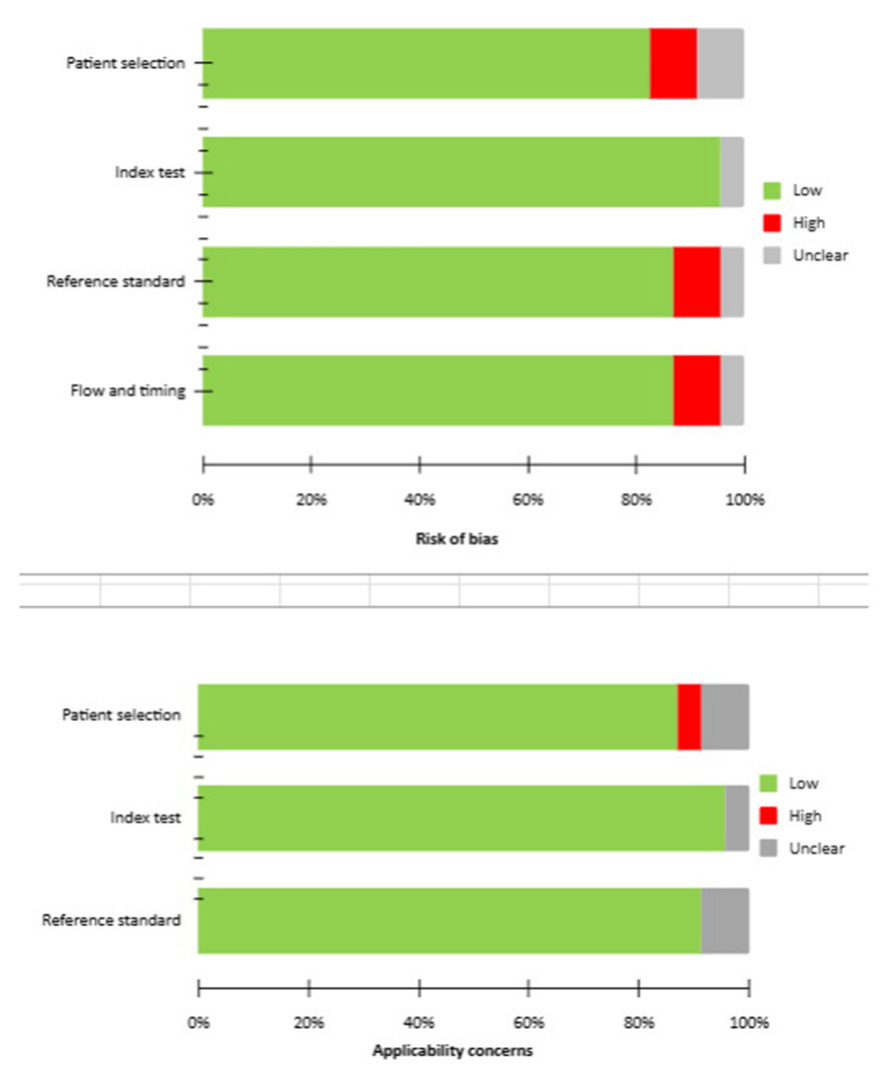
Summary of the quality assessment of the included studies using the QUADAS-2 tool.

The selection process is detailed in Figure 1. An initial search of the PubMed/MEDLINE, Scopus, and Cochrane Library databases yielded 67 records. After screening titles and abstracts, 44 articles were excluded: 23 were outside the scope of this review, 3 were reviews, editorials, or letters, and 18 were case reports or small case series. The full-text versions of the remaining 23 articles were retrieved and assessed (14–36). A manual search of the references of these studies identified no further eligible papers. Consequently, 23 articles were included in the qualitative synthesis. For the quantitative analysis, 4 studies were excluded: 3 due to potential patient population overlap (17, 23, 24) and 1 due to insufficient data for calculation of DR (20). Ultimately, 19 studies were included in the final meta-analysis: 7 studies (18, 27–29, 32, 34, 35) investigated staging SPECT/CT; 9 studies (15, 16, 19, 21, 26, 31, 33, 36) biochemical recurrence and 3 studies both indications (14, 22, 30). Study characteristics and primary outcomes are summarized in Tables 1–4, while the quality assessment of the included studies is presented in Figure 2.

**Table 1:** Studies' general information.

Study	Year	Country	Funding source	Study design	N°patients	Median/ mean age	Median/ mean PSA	Setting
Lawal et al. (14)	2017	South Africa	None declared	P	14	68 (50–81)	45.18 (1.51–687)	Comparison with Ga68-PSMA
Reinfelder et al. (15)	2017	Germany	Molecular insight pharmaceuticals	R	60	71 (49–85)	2.6 (0.2–520.6)	Detection rate in biochemical recurrence
Schmidkonz et al. (16)	2018	Germany	None declared	R	225	72 (50–89)	3.5 (0.01–93)	Detection rate in biochemical recurrence
Schmidkonz et al. (17)	2018	Germany	Molecular insight pharmaceuticals	R	28	(54–84)	(1–178)	Treatment response in metastatic PC
Schmidkonz et al. (18)	2018	Germany	None declared	R	93	65 (51–81)	5.7 (1–742)	Primary staging
Liu et al. (19)	2018	China	None declared	R	208	68 (44–84)	5.4 (0.2–36)	Detection rate in biochemical recurrence
García-Pérez et al. (20)	2018	Mexico	None declared	P	23	68 (55–85)	80.85 (0.38–517)	Comparison with Ga68-PSMA in biochemical recurrence mcrPC
Schmidkonz et al. (21)	2019	Germany	None declared	R	50	69 (52–83)	0.49 (0.2–0.9)	Detection rate in biochemical recurrence (psa < 1)
Werner et al. (22)	2020	Germany	Collaborative Research Center grant of the Deutsche Forschungs-gemeinschaft (SFB824; subproject Z1).	R	210	73	50 (0.01–3500)	Diagnostic use in different settings
Schmidkonz et al. (23)	2020	Germany	None declared	R	22	68 (54–81)	26 (2.4–484)	Interobserver and intraobserver variability in mPC
Schmidkonz et al. (24)	2020	Germany	None declared	R	125	72 (51–88)	3.9 (0.8–18.4)	Treatment response in biochemical recurrence
Sergieva et al. (25)	2021	Bulgaria	None declared	P	36	69.44 (60–80)	6.73 (0.1–73)	Detection rate in biochemical recurrence
Li et al. (26)	2022	China	Henan Key Laboratory of Molecular Nuclear Medicine and Translational Medicine (grant no. 2020-27-4) and Henan Provincial Medical Science and Technology Project (grant no. SBGJ202102015)	R	147	70 (49–87)	8.26 (0.22–187.40)	Detection rate in biochemical recurrence
Wang et al. (27)	2023	China	Shanghai Municipal Health Commission (201840076).	R	31	69.8 (54–88)	62.2 (2.14–147)	Detection rate at staging
Ghaedian et al. (28)	2023	Iran	Shiraz University of Medical Sciences (Grants No. 20293)	P	40	67.7	26.9	Comparison with Tc99m bone scintigraphy in staging
Zhang et al. (29)	2023	China	Startup Fund for scientific research, Fujian Medical University (2021QH1282) and Fujian Provincial Department of Finance (MCZ [2021] No. 0917).	P	56	70 (29–87)	14.8 (5.1–710)	Comparison with mMRI in detection primary prostate cancer
Farkas et al. (30)	2024	Hungary	Not declared	R	100	Staging group 71.5 (66–72) Restaging group 71 (57–86)	Staging group 16.24 (2.73–27.03) Restaging group 3.2 (1.01–9.66)	Detection rate at staging and biochemical recurrence or progressive disease
Aryana et al. (31)	2025	Iran	Not declared	P	39	69.7 (51–87)	271.7	Comparison with Tc99m bone scintigraphy in mCRPC receiving Lu177-PSMA-617
Currie et al. (32)	2025	Australia	Telix Pharmaceuticals	P	27	74 (58–85)	33.1 (0.2–254)	Clinical impact
Taywade et al. (33)	2025	India	Not declared	P	18	nr	85.9	Comparison with Tc99m bone scintigraphy
RoshanRavan et al. (34)	2025	Iran	Mashhad University of Medical Sciences	P	63	68.25 (46–90)	nr	Diagnostic role in staging moderate-high risk
Jonmarker et al. (35)	2026	New Zealand	Not declared	R	82	68.2	19.4	Diagnostic performance compared to MRI in primary staging
Liepe et al. (36)	2026	Germany	Not declared	R	143	73.8 (47–92)	1.45 (0.02–3451.1)	Diagnostic role in biochemical recurrence

R, retrospective; P, prospective; nr, not reported.

### Systematic review (qualitative analysis)

#### Study and patient characteristics

Twenty-three articles evaluating the DR of [^99m^Tc]Tc-PSMA SPECT/CT in PCa patients were selected for a total of 1840 patients (Table 1) (14–36). The selected articles were published in the last nine years by researchers from Europe, Asia, Africa, America, and Oceania; 9 out of 23 studies were prospective studies (39%). Mean and median age of the patients included in these studies ranged from 65 to 74 years. Mean and median PSA serum values before SPECT/CT among the included PCa patients ranged from 0.49 to 85.9 ng/mL. Ten studies have as main endpoints the investigation of DR in biochemical recurrence PCa, while 6 studied the evaluation of DR in staging. The remaining studies had heterogeneous aims.

#### Technical features

Technical aspects of the included studies are reported in Table 2. A total of three radiotracers were included: [^99m^Tc]Tc-MIP-1404 in eight studies; [^99m^Tc]-HYNIC-PSMA in 13 studies; [^99m^Tc]Tc-PSMA I&S in the other 2 studies. The hybrid imaging modality was always SPECT/CT, with different scanners. The injected radiopharmaceutical activity ranged from 555 to 1100 MBq according to local protocol; while uptake time (interval between radiotracer injection and image acquisition) were quite heterogeneous (1–5 h). Analysis of SPECT/CT images was performed using qualitative criteria (visual analysis) in all the studies. A clear reference standard was not specified in the included studies and when reported was very heterogeneous. In most studies a combination of histological and imaging data were used as reference standard.

**Table 2:** Main technical features of the scanner and protocols.

Study	Radiotracer	Type of scanner	Radiotracer activity injected, (MBq)	Uptake time (hours)	Protocol	Comparator
Lawal et al. (14)	Tc-99m HYNIC-PSMA	Infinia Hawkeye dual head hybrid scanner (GE Healthcare, Milwaukee, WI).	1,100	3	Total body scintigraphy + SPECT/CT abdomen-pelvis	Imaging (Ga68-PSMA PET/CT)
Reinfelder et al. (15)	^99m^Tc–MIP-1404	Symbia T2 SPECT/CT system (Siemens Healthcare, Hoffman Estates, IL)	612–842.5	Nr	Total body scintigraphy + SPECT/CT thorax and abdomen	Histological or imaging (CT, MRI and others)
Schmidkonz et al. (16)	^99m^Tc-MIP-1404	Symbia T2	708	3–4	Total body scintigraphy + SPEC/CT thorax and abdomen	Histological
Schmidkonz et al. (17)	^99m^Tc–MIP-1404	Symbia T2 SPECT/CT system (Siemens Healthcare, Hoffman Estates, IL)	712	3–4	Total body scintigraphy + SPECT/CT thorax, abdomen and pelvis	Biochemical response
Schmidkonz et al. (18)	^99m^Tc–MIP-1404	Symbia T2 SPECT/CT system (Siemens Healthcare, Hoffman Estates, IL)	706	3–4	Total body scintigraphy + SPECT/CT thorax and abdomen	Histological
Liu et al. (19)	Tc-99m HYNIC-PSMA	Discovery NM/CT 670 (GE Healthcare, Milwaukee, WI).	728	2	Total body scintigraphy + SPECT/CT	Histological or imaging (CT, FDG PET/CT, bone scintigraphy or MRI)
García-Pérez et al. (20)	Tc-99m HYNIC-PSMA	nr	555–740	3	SPECT/CT skull to thighs	Imaging (Ga68-PSMA PET/CT)
Schmidkonz et al. (21)	^99m^Tc-MIP-1404	Symbia T2	691	3–4	total body scintigraphy + SPEC/CT thorax and abdomen	Histological
Werner et al. (22)	[^99m^Tc]Tc-PSMA-I&S	Optima NM/CT 640 SPECT/CT	761.5	5	Total body scintigraphy + SPECT/CT thorax and abdomen	Nr
Schmidkonz et al. (23)	^99m^Tc–MIP-1404	Symbia T2 SPECT/CT system (Siemens Healthcare, Hoffman Estates, IL)	709	3–4	Total body scintigraphy + SPECT/CT thorax, abdomen and pelvis	Histological
Schmidkonz et al. (24)	^99m^Tc–MIP-1404	Symbia T2 or T6 SPECT/CT system (Siemens Healthcare, Hoffman Estates, IL)	nr	nr	Total body scintigraphy + SPECT/CT thorax, abdomen and pelvis	Histological and CT
Sergieva et al. (25)	Tc-99m HYNIC-PSMA	Symbia T2, Siemens	6.3/Kg	1–3	Total body scintigraphy + SPECT/CT thorax and abdomen	Histological or imaging (CT, bone scintigraphy or MRI)
Li et al. (26)	Tc-99m HYNIC-PSMA	Symbia T16 (Siemens, Germany)	10 MBq/kg	3–4	Total body scintigraphy + SPECT/CT abdomen-pevis +/- thorax	nr
Wang et al. (27)	Tc-99m HYNIC-PSMA	Discovery NM/CT 670, General Electric Medical Systems, Waukesha, WI	740	3–4	Total body scintigraphy + SPECT/CT	Histological or imaging (CT, bone scintigraphy or MRI)
Ghaedian et al. (28)	Tc-99m HYNIC-PSMA	nr	740	3	Total body scintigraphy + SPECT/CT	Histological or imaging (CT, bone scintigraphy or MRI)
Zhang et al. (29)	Tc-99m HYNIC-PSMA	Discovery NM/CT 670Pro	740	2	Total body scintigraphy + SPECT/CT	Histological
Farkas et al. (30)	[^99m^Tc]Tc-PSMA-I&S	Mediso Anyscan TRIO; (Mediso Medical Imaging Systems Ltd, Budapest, Hungary)	666	6	Whole body SPECT/CT	Histological, imaging (CT, bone scintigraphy or MRI) or PSA decrement after radiotherapy
Aryana et al. (31)	Tc-99m HYNIC-PSMA	nr	740	3–5	Total body scintigraphy + SPECT/CT thorax and abdomen	Histological or imaging (CT, bone scintigraphy or MRI)
Currie et al. (32)	Tc-99m HYNIC-PSMA	nr	650–800	2–4	Total body scintigraphy + SPECT/CT thorax and abdomen	Nr
Taywade et al. (33)	Tc-99m HYNIC-PSMA	Discovery NM/CT 670 (GE Healthcare, Milwaukee, WI).	555–740	4	Total body scintigraphy + SPECT/CT	Histological or imaging (CT, bone scintigraphy or MRI), follow-up
RoshanRavan et al. (34)	Tc-99m HYNIC-PSMA	nr	740–925	3–5	Total body scintigraphy + SPECT/CT chest and abdomen and pelvis	Imaging (CT and bone scintigraphy)
Jonmarker et al. (35)	Tc-99m HYNIC-PSMA	Symbia Intevo Bold or Siemens Pro.specta xSPECT X7 (Siemens Healthineers, Erlangen, Germany).	650–700	3–4	Total body scintigraphy + SPECT/CT chest and abdomen	Imaging (MRI)
Liepe et al. (36)	[^99m^Tc]Tc-(CO)3-MIP-1404	GE NM/CT 850	664	1–2.5	SPECT/CT from proximal femora to chest	nr

#### Main findings

Most of the included studies demonstrated a good DR of [^99m^Tc]Tc-PSMA SPECT/CT in PCa patients, both in staging and biochemical recurrent fields. In most studies (15, 16, 18, 19, 21, 26, 27, 30, 34, 36) the DR of [^99m^Tc]Tc-PSMA SPECT/CT was significantly associated with PSA value at the time of the scan. Other features significantly associated with rate of positive SPECT/CT scan were Gleason score (16, 18, 19, 21, 27, 34), the presence of androgen deprivation therapy (16, 21), tumor size (14, 30) and the PSA doubling time (19, 36). Generally there was a direct relationship between PSA value and SPECT positivism: the proportion of positive scans increased with PSA levels. No significant adverse effects of [^99m^Tc]Tc-PSMA SPECT/CT were reported. The change of management by using SPECT/CT in PCa was reported only in one research (32).

Two articles compared the DR of v SPECT/CT with [^68^Ga]Ga-PSMA PET/CT (14, 20) showing a superiority of PET/CT. Three studies compared [^99m^Tc]Tc-PSMA SPECT/CT with Tc99m-bone scan (28, 31, 33). In the first study (28), [^99m^Tc]Tc-PSMA SPECT/CT showed better diagnostic performances than bone scintigraphy with a sensitivity of 83.3% vs. 50%, specificity of 100% vs. 82.1%, and accuracy of 95% vs. 72.5%. In another study (31) focus on Pca patients after radioligand treatment, [^99m^Tc]Tc-PSMA SPECT/CT with Tc99m-bone scan had different behavior: in 13 patients [^99m^Tc]Tc-PSMA SPECT/CT recognized more skeletal lesion than bone scintigraphy; in 12 patients both scans revealed the same lesions; while in the remaining 12 patients Tc99m-bone scan was superior.

Finally, Taywade et al. (33) demonstrated that Tc99m-MDP bone scintigraphy recognized a higher number of skeletal lesions than [^99m^Tc]Tc-PSMA, despite this difference was not statistically significant. However, the authors underlined the advantage of [^99m^Tc]Tc-PSMA to search lesions also in soft tissues and its potential theranostic application.

### Meta-analysis (quantitative analysis)

#### Staging

**Figure 3:**
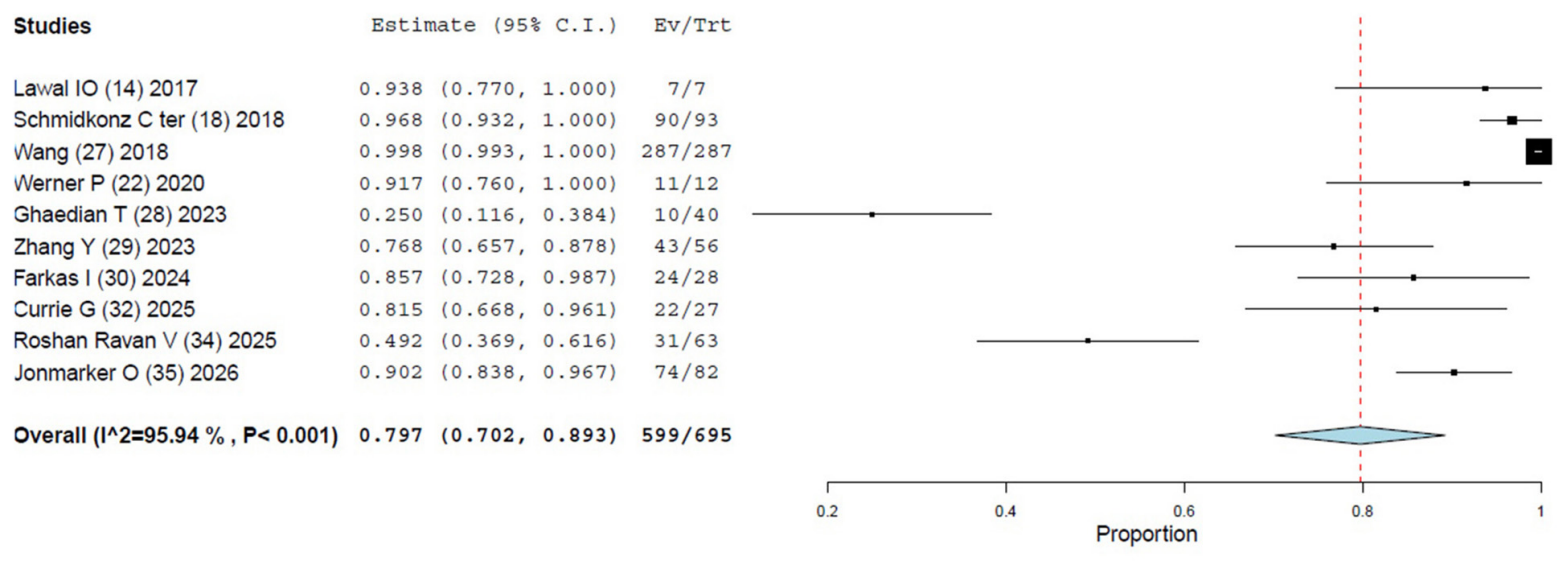
Meta-analysis on the detection rate of [^99m^Tc]Tc-PSMA SPECT/CT in patients with PCa at staging. Meta-analysis was performed using a random-effects model. Pooled values were presented along with corresponding 95% confidence intervals (95% CI) values. The size of the squares indicates the weight of each study.

**Figure 4:**
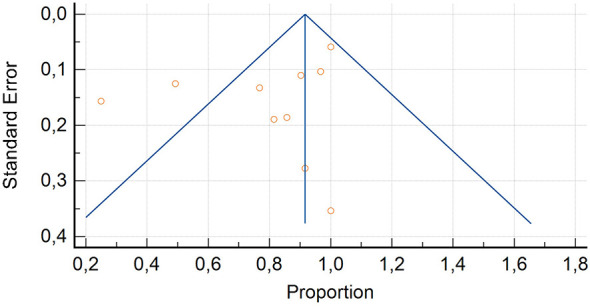
Funnel plot for the evaluation for bias assessment of studies concerning [^99m^Tc]Tc-PSMA SPECT/CT in patients with PCa at staging.

Ten studies were selected (14, 18, 22, 27–30, 32, 34, 35) The overall DR of [^99m^Tc]Tc-PSMA SPECT/CT on a per patient-based analysis ranged from 25% to 100%, and the pooled estimate DR was 79.7% (95% CI 70.2–89.3%) (Table 3, Figure 3). A significant heterogeneity among the included studies was derived (I^2^ = 95.94%). Visual analysis of the funnel plot is presented in Figure 4 and represents a significant heterogeneity. Seven of these ten studies (14, 27–29, 32, 34, 35) used [^99m^Tc]-HYNIC-PSMA as radiopharmaceutical, two studies (22, 30) [^99m^Tc]Tc-PSMA I&S and one research (18) [^99m^Tc]Tc-MIP-1404. To reduce heterogeneity, we conducted a sub-analysis only for studies that used [^99m^Tc]-HYNIC-PSMA but the detection rate remained similar (74.1% 95% CI 56.6–91.5%) and heterogeneity remained very (I^2^ = 97.2%) (Supplementary Figure 1A). Also a sub-analysis including only prospective studies (14, 28, 29, 32, 34) derived lower detection rate (65% 95% CI 41.7–88.3%) and high heterogeneity (I^2^ = 93.4%) (Supplementary Figure 1B).

**Table 3:** Diagnostic performances in a per-patient analysis in staging field.

Study	Year	N°patients	Detection rate	Factors correlated with detection rate
Lawal et al. (14)	2017	7	7/7 (100%)	Tumor size
Schmidkonz et al. (18)	2018	93	90/93 (97%)	PSA level, Gleason score
Wang et al. (27)	2018	287	287/287 (100%)	PSA level, Gleason score
Werner et al. (22)	2020	12	11/12 (92%)	nr
Ghaedian et al. (28)	2023	40	10/40 (25%)	nr
Zhang et al. (29)	2023	56	43/56 (77%)	nr
Farkas et al. (30)	2024	28	24/28 (86%)	PSA level
Currie et al. (32)	2025	27	22/27 (81.5%)	nr
RoshanRavan et al. (34)	2025	63	31/63 (49%)	PSA level, Gleason score
Jonmarker et al. (35)	2026	82	74/82 (90%)	nr

Nr, not reported.

#### Biochemical recurrence

**Figure 5:**
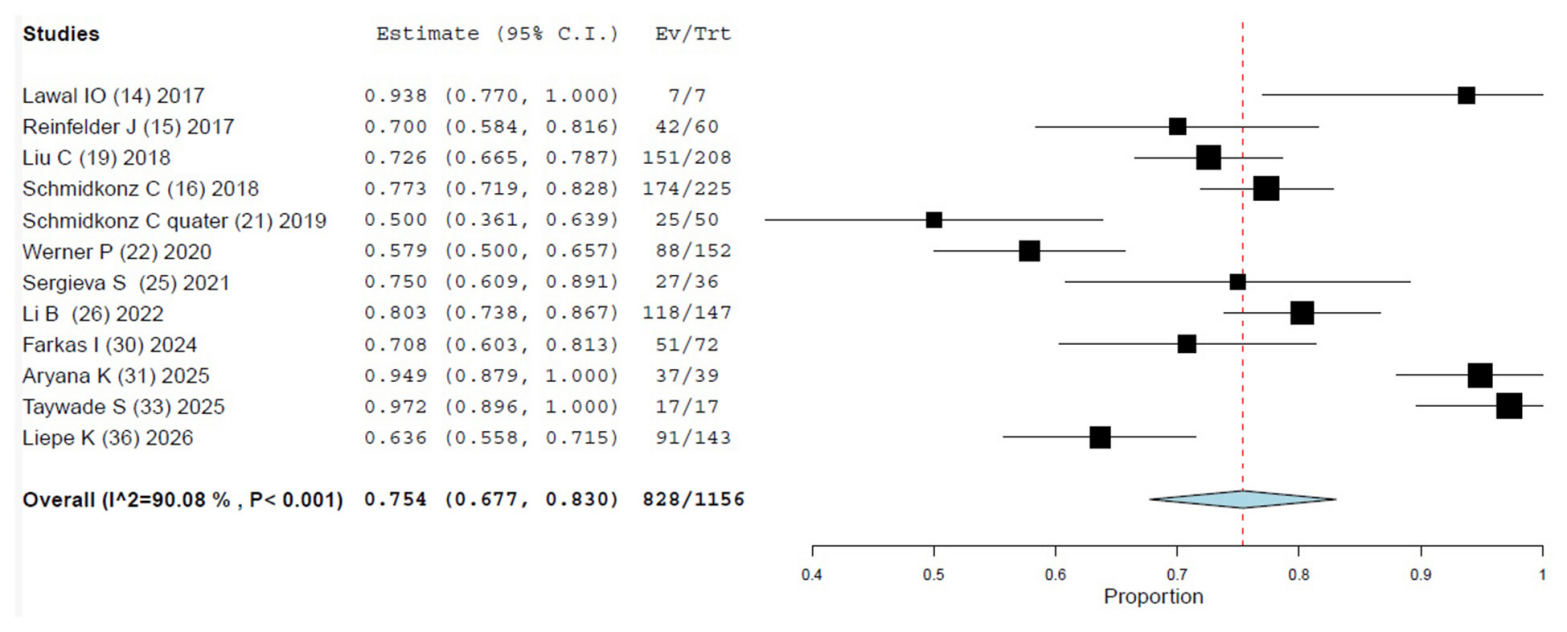
Meta-analysis on the detection rate of [^99m^Tc]Tc-PSMA SPECT/CT in patients with PCa at biochemical recurrence. Meta-analysis was performed using a random-effects model. Pooled values were presented along with corresponding 95% confidence intervals (95% CI) values. The size of the squares indicates the weight of each study.

**Figure 6:**
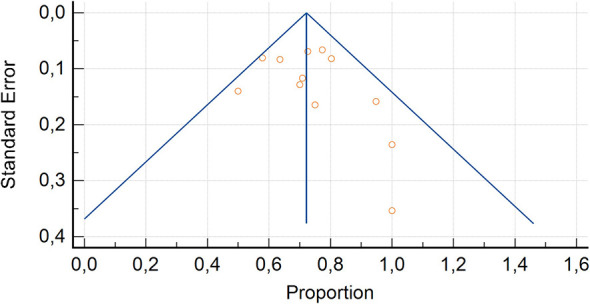
Funnel plot for the evaluation for bias assessment of studied concerning [^99m^Tc]Tc-PSMA SPECT/CT in patients with PCa at biochemical recurrence.

Twelve studies were selected (14–16, 19, 21, 22, 25, 26, 30, 31, 33, 36) The overall DR of [^99m^Tc]Tc-PSMA SPECT/CT on a per patient-based analysis ranged from 50% to 100%, and the pooled estimate DR was 75.4% (95%CI 67.7–83%) (Table 4, Figure 5). A significant heterogeneity among the included studies was derived (I^2^ = 90.04%). Visual analysis of the funnel plot is presented in Figure 6 and represents a significant heterogeneity. Six studies (14, 19, 25, 26, 31, 33) used [^99m^Tc]-HYNIC-PSMA as radiopharmaceutical, four studies (15, 16, 21, 36) [^99m^Tc]Tc-MIP-1404 and one research (22) [^99m^Tc]Tc-PSMA I&S. A sub-analysis only for studies that used [^99m^Tc]-HYNIC-PSMA showed a better detection rate of 83.4% (95% CI 74.6–92.3%) but always a significant heterogeneity (I^2^ = 86.58%) (Supplementary Figure 2A); a sub-analysis for [^99m^Tc]Tc-MIP-1404 demonstrated a lower DR of 66.3% (95% CI 55.5–77.1%) and a similar heterogeneity (I^2^ = 82.67%) (Supplemental Figure 2B). Instead a subsequent analysis based on nature of the study (prospective vs. retrospective) derived a low DR for retrospective studies 68.7% (95% CI 62.3–75.1%) and I^2^ = 80% (Supplementary Figure 3A), while for prospective studies the DR was excellent 91.7% (95% CI 83.4–99.9%) and not significant heterogeneity (I^2^ = 60.76%) (Supplementary Figure 3B).

**Table 4:** Diagnostic performances in a per-patient analysis in the restaging field of biochemical recurrence.

Study	Year	N°patients	Detection rate	Factors correlated with detection rate
Lawal et al. (14)	2017	7	7/7 (100%)	Tumor size
Reinfelder et al. (15)	2017	60	42/60 (70%)	PSA level
Liu et al. (19)	2018	208	151/208 (73%)	PSA level, PSA doubling time, Gleason score
Schmidkonz et al. (16)	2018	225	174/225 (77%)	PSA level, Gleason score, androgen deprivation therapy
Schmidkonz et al. (21)	2019	50	25/50 (50%)	PSA level, Gleason score, androgen deprivation therapy
Werner et al. (22)	2020	152	88/152 (58%)	PSA
Sergieva et al. (25)	2021	36	27/36 (75%)	nr
Li et al. (26)	2022	147	118/147 (80%)	PSA level
Farkas et al. (30)	2024	72	51/72 (71%)	PSA level, tumor size
Aryana et al. (31)	2025	39	37/39 (95%)	nr
Taywade et al. (33)	2025	17	17/17 (100%)	nr
Liepe et al. (36)	2026	143	91/143 (64%)	PSA level, PSA doubling time, ISUP

nr, not reported.

## Discussion

The current systematic review and meta-analysis aimed to evaluate the diagnostic performance of [^99m^Tc]Tc-PSMA SPECT/CT in patients with prostate cancer (PCa), focusing on its utility in both primary staging and the detection of biochemical recurrence (BCR). Our findings suggest that [^99m^Tc]Tc-PSMA SPECT/CT is a good diagnostic tool with pooled detection rates (DR) of 79.7% for primary staging and 75.4% for biochemical recurrence. The results demonstrate that [^99m^Tc]Tc-PSMA SPECT/CT maintains a high detection rate across different disease stages. In the setting of BCR, the DR was significantly associated with PSA levels at the time of the scan, showing a direct relationship where the proportion of positive scans increased alongside rising PSA values. This mirrors the behavior of PET-based PSMA ligands, which are currently considered the gold standard. Furthermore, other clinical factors such as Gleason score, PSA doubling time, and tumor size were identified as significant predictors of a positive SPECT/CT scan. These variables were demonstrated as predictive factors also of PSMA PET/CT findings (37–39).

A meta-analysis by Yu et al. (40) showed that the sensitivity and specificity of [^68^Ga]Ga-PSMA PET/CT for the diagnosis of prostate cancer were 0.916 (95% CI, 0.896–0.934) and 0.734 (95% CI, 0.685–0.779), respectively, rates higher than those derived by our study. Focus on BCR, the pooled DR of ^18^F-labeled PSMA PET/CT was 81% (95% CI: 71–88%) (41).

While PSMA PET/CT remains superior in head-to-head comparisons due to its higher spatial resolution and sensitivity, [^99m^Tc]Tc-PSMA SPECT/CT offers distinct advantages. Compared to conventional Tc99m-MDP bone scans, PSMA-targeted SPECT/CT generally showed better diagnostic performance and accuracy and the big advantage to investigate skeletal and not-skeletal lesions at the same time. This allows a more comprehensive whole-body assessment and subsequent management.

However, the literature remains somewhat divided on skeletal detection; while some studies found SPECT/CT superior, others noted that bone scans might still recognize a higher number of skeletal lesions in specific contexts, though often without statistical significance.

A critical takeaway from this analysis is the potential for [^99m^Tc]Tc-PSMA SPECT/CT to bridge the diagnostic gap in regions with limited resources (42). The reliance on cyclotrons for ^18^F or generators for ^68^Ga limits PET/CT availability in many low-to-middle-income countries. In contrast, Tc99m is the most widely available radionuclide globally, characterized by lower costs compared to positron emitters; lower radiation burden for patients and favorable physical characteristics for use with existing SPECT/CT infrastructure. While PET/CT remains the preferred choice when available, [^99m^Tc]Tc-PSMA SPECT/CT represents a valuable and cost-effective alternative that could significantly improve the staging and restaging of PCa patients in areas where PET technology is inaccessible.

The meta-analysis revealed significant statistical heterogeneity (I^2^ values of 96.38% for staging and 88.4% for BCR). This variability likely stems from the heterogeneity of the included studies, which used different radiotracers (Tc-MIP-1404, HYNIC-PSMA, and Tc-PSMA I&S), varying uptake times (1–5 h), and different injected activities. While all target the PSMA receptor, their chemical heterogeneity may introduce variability that is difficult to isolate without head-to-head comparative trials. However, in the biochemical recurrence field, [^99m^Tc]-HYNIC-PSMA seems to have a better DR than Tc-MIP-1404 (83.6 vs. 66.4%). A comparative analysis within the staging setting was not feasible, as the available data relied almost exclusively on [^99m^Tc]-HYNIC-PSMA. Additionally, the lack of a standardized reference standard across studies—often relying on a combination of imaging and histology—may contribute to the observed bias. Moreover, each study is deeply heterogeneous. For example in the study of Schmidkonz (21), only patients with suspected recurrence and low PSA (< 1 ng/ml) were included.

The evolving landscape of prostate cancer imaging suggests that [^99m^Tc]Tc-PSMA SPECT/CT will play an increasingly strategic role, particularly in enhancing global health equity. Future research and clinical implementation should focus on the standardization of the protocols. For this reason future multicenter prospective trials are needed to establish standardized acquisition parameters and universal qualitative or semi-quantitative criteria for image interpretation. This standardization will be crucial for ensuring consistent diagnostic accuracy across different healthcare systems.

[^99m^Tc]Tc-PSMA SPECT/CT represents a valuable and pragmatic alternative to PET/CT in regions where the latter is unavailable or cost-prohibitive (43). While it shows promise in expanding access to PSMA-targeted imaging, further prospective trials and head-to-head comparisons with PET-based tracers are required before it can be considered a primary diagnostic modality in standard clinical guidelines. Future efforts should focus on technical training and the development of local radiopharmaceutical production to facilitate this integration.

Beyond its diagnostic utility, the potential application of [^99m^Tc]Tc-PSMA as a theranostic “scouting agent” remains an intriguing hypothesis-generating concept (44). Although our data focuses on detection yield, it is possible to speculate that SPECT/CT could, in the future, play a role in radioligand therapy planning or preliminary dosimetry, particularly in settings where PET/CT is unavailable. However, it must be emphasized that our current results do not directly support clinical primacy in this area. Future prospective trials are strictly necessary to determine whether [^99m^Tc]Tc-PSMA SPECT/CT can reliably predict treatment response or assist in complex dosimetric calculations as a pragmatic alternative to PET-based planning.

Finally, the integration of artificial intelligence (AI) and radiomics into SPECT/CT analysis may help overcome some of the inherent spatial resolution limitations of SPECT (45). Machine learning algorithms trained on large datasets could improve the detection of small-volume nodal disease and better differentiate between benign uptake and true malignancy.

## Data Availability

The original contributions presented in the study are included in the article/Supplementary material, further inquiries can be directed to the corresponding author.
