# Supplementary figures and images for: Detection rate of [99mTc]Tc-PSMA SPECT/CT in prostate cancer: a systematic review and a meta-analysis
Source: Front Med (Lausanne). 2026 Apr 13;13:1827510. doi: 10.3389/fmed.2026.1827510 (PMC13111079; doi:10.3389/fmed.2026.1827510)

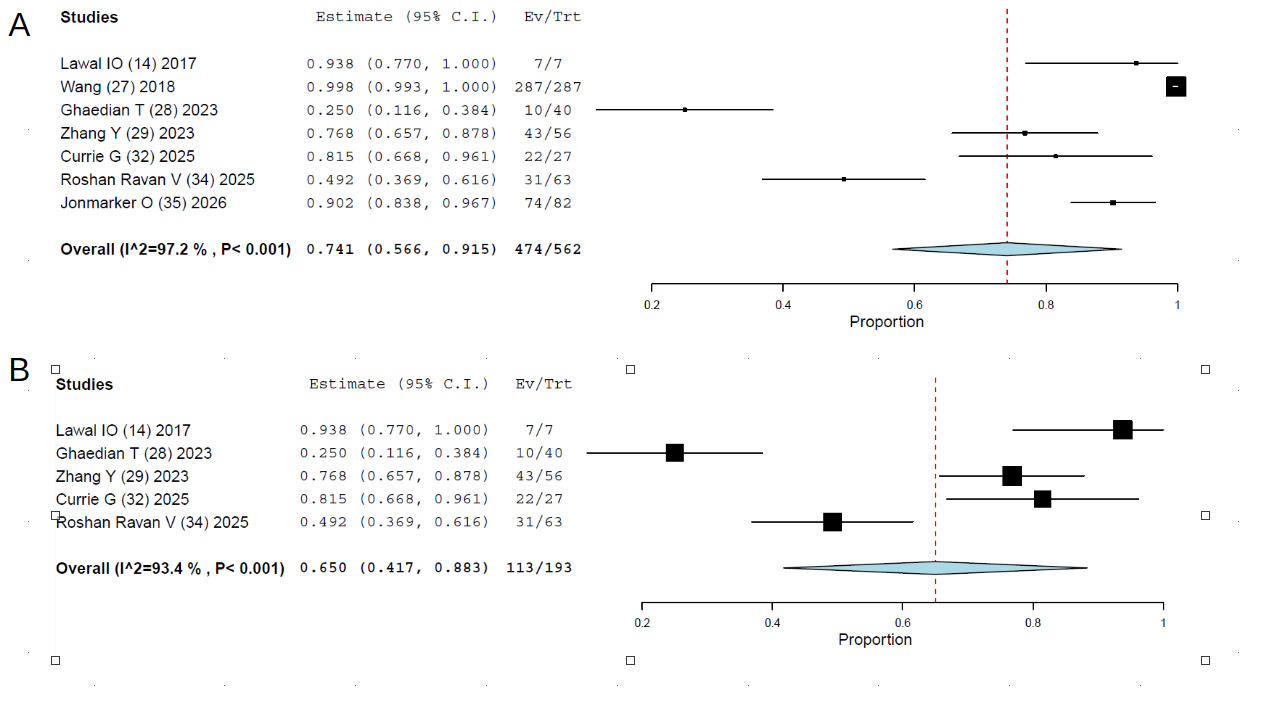

Supplement: Supplementary file 2 [file Image_1.jpeg]

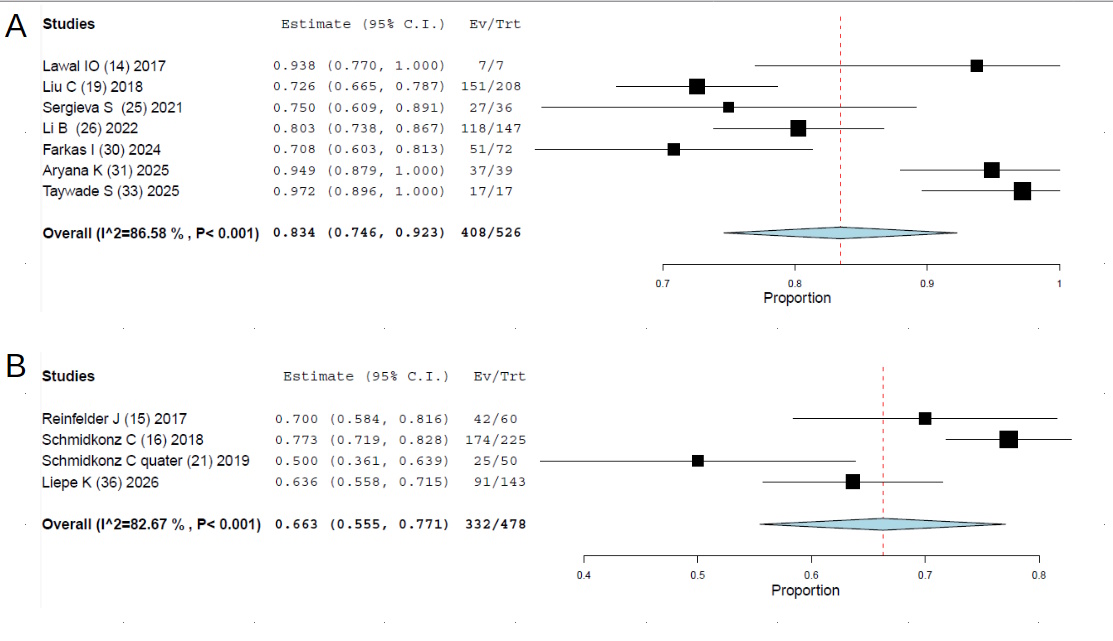

Supplement: Supplementary file 3 [file Image_2.jpeg]

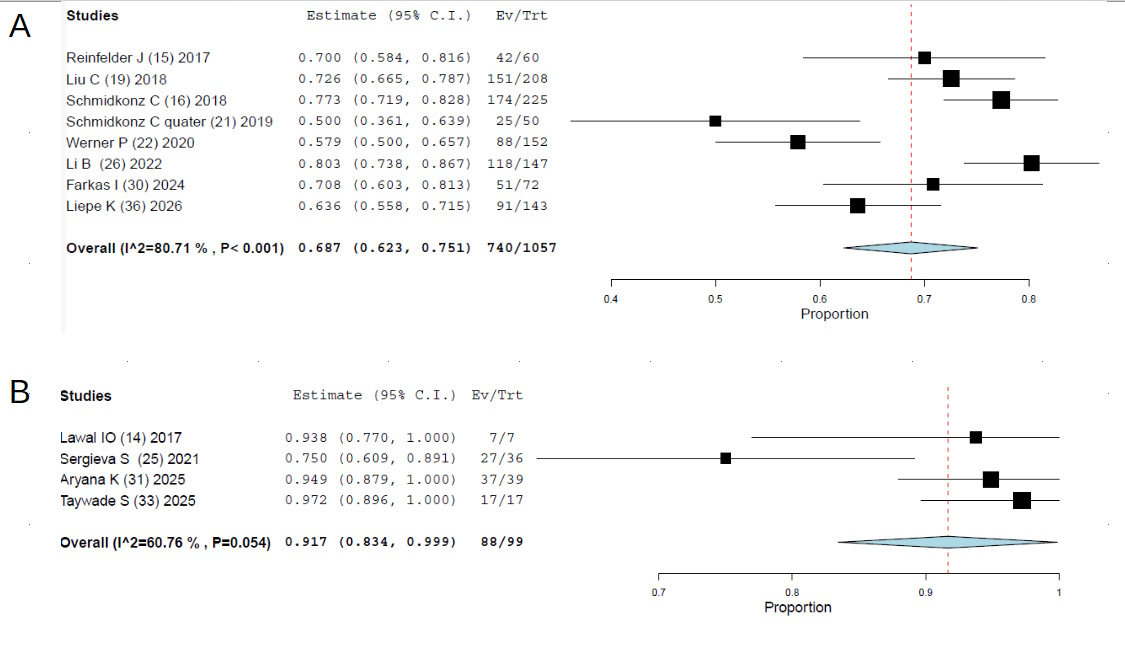

Supplement: Supplementary file 4 [file Image_3.jpeg]
